# OPCABG for Moderate CIMR in Elderly Patients: a Superior Option?

**DOI:** 10.21470/1678-9741-2017-0114

**Published:** 2018

**Authors:** Amber Malhotra, Chandrasekaran Ananthanarayanan, Vivek Wadhawa, Sumbul Siddiqui, Pranav Sharma, Kartik Patel, Komal Shah, Pratik Shah

**Affiliations:** 1 Department of Cardiovascular and Thoracic Surgery of the U. N. Mehta Institute of Cardiology and Research Center (affiliated to BJ Medical College, Ahmedabad), Gujarat, India.; 2 Department of Research of the U. N. Mehta Institute of Cardiology and Research Center (affiliated to BJ Medical College, Ahmedabad), Gujarat, India.

**Keywords:** Mitral Valve Insufficiency/surgery, Mitral Valve Annuloplasty, Mitral Valve/surgery, Coronary Artery Bypass, Coronary Artery Bypass, Off-Pump

## Abstract

**Objective:**

To compare the early and late outcomes of off-pump coronary artery bypass grafting and coronary artery bypass graft + mitral valve repair in elderly patients with moderate chronic ischemic mitral regurgitation.

**Methods:**

One hundred and fifty elderly (age > 70 years) patients with moderate chronic ischemic mitral regurgitation who underwent off-pump coronary artery bypass grafting (n=95) or coronary artery bypass graft + mitral valve repair (n=55) between January 2007 and December 2014 were studied. They were subdivided according to presence or absence of high operative risk. Peri-operative variables and early operative outcomes were retrospectively studied. Survival, mitral regurgitation grade, and functional outcomes were prospectively analysed.

**Results:**

Both groups were comparable in terms of age (*P*=0.23), sex (*P*=0.74), left ventricle ejection fraction (*P*=0.6) and preoperative functional class (*P*=0.52). The mean number of grafts for off-pump coronary artery bypass grafting group was 3.14 and coronary artery bypass graft + mitral valve repair was 3.21. Off-pump coronary artery bypass grafting group had statistically significant better early operative outcomes *i.e* perioperative blood transfusions, intraaortic balloon pump usage, arrhythmias, renal dysfunction, liver dysfunction, sepsis, mean hours of ventilation, intensive care unit stay and operative mortality. On a prospective follow up of 5±2.33 years (1-9 years), coronary artery bypass graft + mitral valve repair in low operative risk subgroup had better improvements in mitral regurgitation grade than off-pump coronary artery bypass grafting. Both groups had similar improvements in functional class and cumulative survival was also comparable (63.2% *vs.* 54.5%).

**Conclusion:**

Off-pump coronary artery bypass grafting is a safer alternative to coronary artery bypass graft + mitral valve repair with better early operative outcomes and comparable late survival and functional outcomes in elderly patients with moderate chronic ischemic mitral regurgitation, especially those with higher operative risk.

**Table:** 

Abbreviations, acronyms & symbols	
ACC	= American College of Cardiology
AHA	= American Heart Association
ASE	= American Society of Echocardiography
CABG	= Coronary artery bypass grafting
CAD	= Coronary artery disease
CIMR	= Chronic ischemic mitral regurgitation
IABP	= Intra-aortic balloon pump
ICU	= Intensive care unit
LV	= Left ventricle
MVRep	= Mitral valve repair
NYHA	= New York Heart Association
OPCABG	= Off-pump coronary artery bypass grafting
RIME	= Randomized ischemic mitral evaluation

## INTRODUCTION

The number of elderly patients undergoing coronary artery bypass grafting (CABG) is on the rise. The continuing improvements in myocardial infarction management, timely thrombolysis and primary percutaneous intervention techniques have saved many lives. The age of patients presenting for CABG has increased and they have very advanced disease, both anatomically and functionally. Elderly patients have a higher burden of surgical risk factors and reduced functional capacity compared to the young. The prevalence of comorbidities such as cerebrovascular diseases, diabetes mellitus, chronic obstructive pulmonary disease, renal dysfunction and peripheral arterial disease are higher in the elderly. Age is a risk factor for poor operative outcome and elderly patients have worse early evolution in comparison to the young^[1]^.

Optimal management strategy of moderate chronic ischemic mitral regurgitation (CIMR) has long remained controversial. It is now well understood that CIMR is a bad prognostic indicator in coronary artery disease (CAD). Mitral regurgitation begets more mitral regurgitation leading to worsening of mitral regurgitation grade and early progression to left ventricle (LV) dysfunction. Several studies have shown the association of CIMR as a negative determinant of survival in patients with CAD^[2-4]^. It is now an accepted strategy to surgically address severe CIMR and to do revascularization alone for mild CIMR at the time of myocardial revascularization. Management of moderate CIMR at the time of CABG is a controversial topic and data regarding the same in elderly is scanty. The addition of mitral procedure in this subset is a difficult decision because of added morbidity of cardiopulmonary bypass and cardioplegic arrest for repair. All the studies on chronic CIMR have compared on-pump CABG and CABG + Mitral valve repair (MVRep). Off pump coronary artery bypass grafting (OPCABG) has proven to be a safer alternative to on pump CABG for high risk patients^[5]^ and this has been studied by many investigators^[6,7]^. This study aims at comparing the outcomes of OPCABG and CABG+MVRep in the setting of moderate CIMR in elderly.

## METHODS

### Study Design

Combined retrospective and prospective observational study.

### Inclusion Criteria

All patients (age > 70 years) who underwent surgery for CAD with moderate CIMR during the study period (January 2007 to December 2014) were included in the study.

### Exclusion Criteria

Patients having any evidence of structural (chordal or leaflet) mitral valve disease and endocarditis were excluded from the study. Patients who underwent surgery in extremis or had an emergency surgery were excluded from the study.

### Study Groups

Of the 150 patients, 95 (63.33%) underwent OPCABG (group I) and the remaining 55 (36.67%) underwent CABG+MVRep (group II).

### Subgroups

Both the groups were divided into two subgroups A & B according to the presence or absence of high operative risk, respectively.

High operative risk is defined as the presence of left ventricular ejection fraction < 30% and one or more of the following risk factors - preoperative renal dysfunction, previous stroke and chronic obstructive pulmonary disease.

### Surgical Procedures

All patients underwent myocardial revascularization, which included a left internal mammary artery graft to left anterior descending coronary artery and great saphenous vein graft for other diseased coronary arteries. Group I underwent OPCABG & group II underwent CABG+MVRep with cardiopulmonary bypass support. In the patients who underwent MVRep, the mitral valve was inspected to rule out any structural lesion. MVRep was done using undersized complete semi-rigid ring (Carpentier-Edwards Physio I annuloplasty ring, Edwards Lifesciences, Irvine, CA, USA). All patients had a successful repair which was defined as less than mild mitral regurgitation at the end of the procedure.

### Collection of Data

All the relevant preoperative, intra-operative and postoperative data were collected from the respective case files after acquiring permission from the institute's ethics committee. They were tabulated for comparison of the subgroups. After acquiring written informed consent, the study cohort was followed up and their functional class, mitral regurgitation grade and survival were recorded.

### Definitions

The definition of moderate ischemic mitral regurgitation followed the guidelines of the American College of Cardiology (ACC), the American Heart Association (AHA), and the American Society of Echocardiography (ASE), and was defined as an effective regurgitant orifice area of 0.20 to 0.39 cm^2^, a regurgitant volume of 30 to 59 mL per beat, a regurgitant fraction of 30% to 49%, or a vena contracta width of 0.30 to 0.69 cm^[8]^.

1. Postoperative renal dysfunction: creatinine > 2mg/dl
2. Postoperative liver dysfunction: liver enzymes & bilirubin > three times the upper limit of normal value.
3. Sepsis: presence of positive blood culture with or without fever and raised white blood cell counts.
4. Arrhythmia: any supraventricular or ventricular arrhythmia.

### Statistical Analysis

The statistical calculations were performed using SPSS software v 20.0 (Chicago, IL, USA) Quantitative data was expressed as mean ± SD whereas qualitative data was expressed as a percentage. Uni-variate analysis of the continuous data was performed using Student's t-test for parametric and Mann-Whitney U-test for non-parametric variables, whereas chi-square test was used for the categorical data. The cut-off value of *P*<0.05 was considered for the statistical significance. Survival analysis was performed using Kaplan Meier.

## RESULTS

### Baseline Characteristics

The mean age of the entire cohort was 76.1 years. Demographic and preoperative clinical characteristics of the study population are presented in Table 1. Both groups were comparable in terms of age (*P*=0.23), sex (*P*=0.74), haemoglobin (*P*=0.2), extent of CAD (*P*=0.27), left ventricular ejection fraction (*P*=0.6), comorbidities - previous stroke (*P*=0.96), diabetes mellitus (*P*=0.11), hypertension (*P*=0.85), recent myocardial infarction (*P*=0.19), chronic obstructive pulmonary disease (*P*=0.69) and renal dysfunction (*P*=0.09) (Table 1).

**Table 1:** Preoperative variables comparison.

Variables		OPCABG	CABG+MVRep	*P* value
		N=95 (63.33%)	N=55 (36.67%)	
Age (years)		76.32±2.74	75.79±2.32	0.23
Sex (male)		67 (70.53%)	41 (74.55%)	0.74
Diabetes		36 (37.89%)	13 (23.64%)	0.11
Hypertension		61 (64.21%)	37 (67.27%)	0.85
COPD		32 (33.68%)	16 (29.09%)	0.69
Recent MI		13 (13.68%)	13 (23.64%)	0.19
Previous stroke		4 (4.21%)	3 (5.45%)	0.96
SVD		27 (28.42%)	16 (29.09%)	0.93
DVD		32 (33.68%)	13 (23.64%)	0.27
TVD		19 (20%)	19 (34.55%)	0.08
LMCA		44 (46.32%)	16 (29.09%)	0.06
Hb (gm %)		11.83±1.64	11.5±1.2	0.2
Creatinine (mg/dl)		1.18 ±0.59	1.24±0.4	0.51
Preoperative LVEF (%)		35.86±13.25	37.06±13.38	0.6
Renal dysfunction		17 (17.89%)	8 (14.55%)	0.09
NYHA	I	__	__	
	II	60 (63.16%)	31 (56.36%)	0.52
	III	35 (36.84%)	24 (43.64%)	0.52
	IV	__	__	

CABG=Coronary artery bypass grafting; COPD=Chronic obstructive pulmonary disease; DVD=Double vessel disease; Hb=Hemoglobin; OPCABG=Off-pump coronary artery bypass grafting. LMCA=Left main coronary artery; LVEF=Left ventricle ejection fraction; MI=Myocardial infarction; MVRep=Mitral valve repair; NYHA=New York Heart Association; SVD=Single vessel disease; TVD=Triple vessel disease

### Operative Variables

All patients underwent complete coronary revascularization. The mean number of grafts in the OPCABG group was 3.14 and in the CABG+MVRep group was 3.21. The mean cardiopulmonary bypass time and mean aortic cross-clamp time in group II were 96.67±20.25 and 66.33±13.14 minutes, respectively.

### Early Post-Operative Outcome

#### Comparison Between Group I and II

Irrespective of the operative risk, CABG+MVRep group (II) had higher incidence of early postoperative complications as compared to OPCABG group (I) (Table 2). The mean number of blood units transfused [I - 2.2±0.5 *vs.* II - 2.9±1.1 (*P*<0.001)], intra-aortic balloon pump (IABP) usage [I - 8.42% *vs.* II - 36.36% (*P*<0.001)], renal dysfunction [I - 8.42 % *vs.* II - 34.55% (*P*<0.001)], arrhythmias [I - 8.42% *vs.* II - 27.27% (*P*=0.004)], liver dysfunction [I - 6.32% *vs.* II - 21.82% (*P*=0.01)], new onset stroke [I - 5.26% *vs.* II -23.64% (*P*=0.002)], sepsis [I - 6.32% *vs.* II - 23.64% (*P*=0.005)], mean duration of ventilation in hours [I - 6.92±2.36 *vs.* II - 17.35±4.66 (*P*<0.001)] and intensive care unit (ICU) stay in days [I - 2.86±1.26 *vs.* II - 5.87±1.98 (*P*<0.001)], were significantly more in the CABG+MVRep group. The thirty-day mortality was also high in CABG+MVRep group [I - 2.11% *vs.* II - 20% (*P*<0.001)] (Table 2).

**Table 2:** Early operative outcome comparison between group I and group II.

Variables	OPCABG N=95 (63.33%)	CABG+MVRep N=55 (36.67%)	*P* Value
Blood transfusion	2.2±0.5	2.9±1.1	<0.001
Postoperative IABP	8 (8.42%)	20 (36.36%)	<0.001
Renal dysfunction	8 (8.42%	19 (34.55%)	<0.001
Arrhythmia	8 (8.42%)	15 (27.27%)	0.004
Liver dysfunction	6 (6.32%)	12 (21.82%)	0.01
New onset stroke	5 (5.26%)	13 (23.64%)	0.002
Sepsis	6 (6.32%)	13 (23.64%)	0.005
30-day mortality	2 (2.11%)	11 (20%)	<0.001
Ventilation (hours)	6.92±2.36	17.35±4.66	<0.001
ICU stay (days)	2.86±1.26	5.87±1.98	<0.001

CABG=Coronary artery bypass grafting; IABP=Intra-aortic balloon pump; ICU=intensive care unit; MVRep=Mitral valve repair; OPCABG=Off-pump coronary artery bypass grafting

#### Subgroup Comparison - High Operative Risk (IA vs. IIA)

OPCABG patients had better early operative outcomes in comparison with the CABG+MVRep patients in all the parameters studied (Table 3). The mean number of blood units transfused [IA - 2.3±0.6 *vs.* IIA - 3.1±0.9 (*P*<0.001)], IABP usage [IA - 8.57% *vs.* IIA - 50% (*P*=0.002)] renal dysfunction [IA - 11.43% *vs.* IIA - 45% (*P*=0.01)], arrhythmias [IA - 11.43% *vs.* IIA - 40% (*P*=0.03)], liver dysfunction [IA - 8.57% *vs.* IIA - 35% (*P*=0.04)], new onset stroke [IA - 5.71% *vs.* IIA - 30% (*P*=0.04)], sepsis [IA - 8.57% *vs.* IIA ± 2.45 *vs.* IIA - 18.24±4.72 (*P*<0.001)] and ICU stay in days [IA - 3.63±1.12 *vs.* IIA - 6.42±2.1 (*P*<0.001)], were significantly more in the CABG+MVRep group. The thirty-day mortality was also high in CABG+MVRep subgroup [IA - 2.86% *vs.* IIA - 25% (*P*=0.04)] (Table 3).

**Table 3:** Early operative outcome comparison in high operative risk subgroup (A).

Variables	IA - OPCABG N=35	IIA- CABG+MVRep N=20	*P* Value
Blood transfusion	2.3±0.6	3.1±0.9	<0.001
Postoperative IABP	3 (8.57%)	10 (50%)	0.002
Renal dysfunction	4 (11.43%)	9 (45%)	0.01
Arrhythmia	4 (11.43%)	8 (40%)	0.03
Liver dysfunction	3 (8.57%)	7 (35%)	0.04
New-onset stroke	2 (5.71%)	6 (30%)	0.04
Sepsis	3 (8.57%)	7 (35%)	0.04
30-day mortality	1 (2.86)	5 (25%)	0.04
Ventilation (hours)	7.23±2.45	18.24±4.72	<0.001
ICU stay (days)	3.63±1.12	6.42±2.1	<0.001

CABG=Coronary artery bypass grafting; IABP=Intra-aortic balloon pump; ICU=Intensive care unit; MVRep=Mitral valve repair; OPCABG=Off-pump coronary artery bypass grafting

#### Subgroup Comparison - Low Operative Risk (IB vs. IIB)

Even in the low operative risk subgroup, OPCABG patients had significantly lower incidence of blood units transfused [IB - 2.1±0.5 *vs.* IIB - 2.7±1.5 (*P*=0.005)], IABP usage [IB - 8.33% *vs.* IIB - 28.57% (*P*=0.02)], renal dysfunction [IB - 6.67% *vs.* IIB - 28.57% (*P*=0.001)], mean duration of ventilation in hours [IB - 6.76±2.25 *vs.* IIB - 15.76±4.13 (*P*<0.001)] and ICU stay in days [IB - 2.78±1.34 *vs.* IIB - 5.38±1.92 (*P*<0.001)] than the CABG+MVRep patients. The thirty-day mortality was also low in OPCABG subgroup [IB - 1.67% *vs.* IIB - 17.14% (*P*=0.01)]. Other parameters were not significantly different between the subgroups (Table 4).

**Table 4:** Early operative outcome comparison in low operative risk subgroup (B).

Variables	IB - OPCABG N=60	IIB- CABG+MVRep N=35	*P* Value
Blood transfusion	2.1±0.5	2.7±1.5	0.005
Postoperative IABP	5 (8.33%)	10 (28.57%)	0.02
Renal dysfunction	4 (6.67%)	10 (28.57%)	0.01
Arrhythmia	4 (6.67%)	7 (20%)	0.1
Liver dysfunction	3 (5%)	5 (14.29%)	0.23
New-onset stroke	3 (5%)	7 (20%)	0.05
Sepsis	3 (5%)	6 (17.14%)	0.11
30-day mortality	1 (1.67%)	6 (17.14%)	0.01
Ventilation (hours)	6.76±2.25	15.76±4.13	<0.001
ICU stay (days)	2.78±1.34	5.38±1.92	<0.001

CABG=Coronary artery bypass grafting; IABP=Intra-aortic balloon pump; ICU=intensive care unit; MVRep=Mitral valve repair; OPCABG=Off-pump coronary artery bypass grafting

### Follow-Up Study

In a mean follow-up period of five years, both OPCABG and the CABG+MVRep group had improvement in the mitral regurgitation grade and New York Heart Association (NYHA) class.

### Improvement in Mitral Regurgitation Grade

**Fig. 1:**
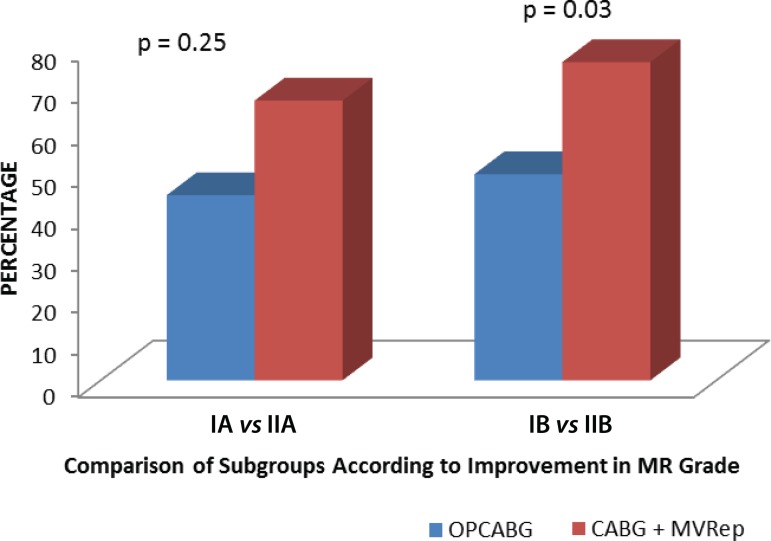
Follow-up comparison of mitral regurgitation (MR) grade between the subgroups. CABG=coronary artery bypass grafting; MVRep=mitral valve repair; OPCABG=off-pump coronary artery bypass grafting

CABG+MVRep was able to offer better results in terms of mitral regurgitation reduction in the low operative risk subgroup, however the results are comparable in the high operative risk subgroup (Figure 1).

### Functional Class Improvement

**Fig. 2:**
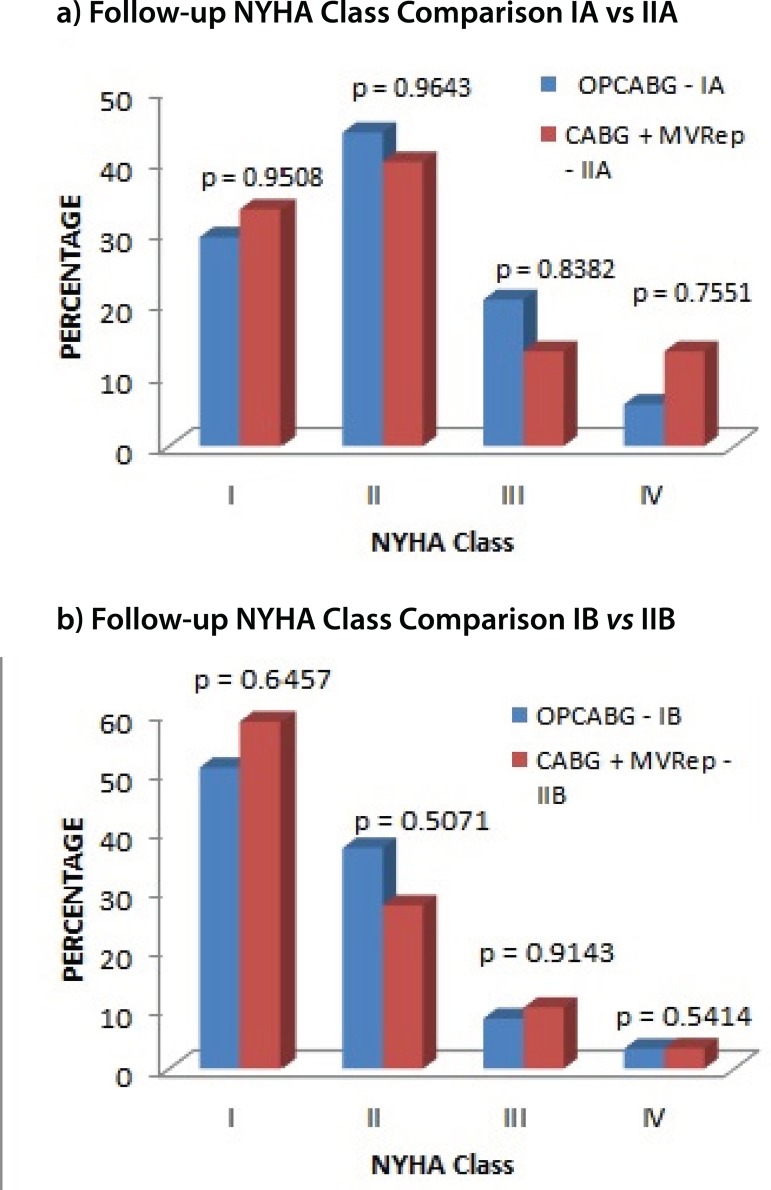
Follow-up comparison of New York Heart Association (NYHA) class between the subgroups. CABG=coronary artery bypass grafting; MVRep=mitral valve repair; OPCABG=off-pump coronary artery bypass grafting

In a mean follow up period of five years, both groups had comparable improvements in their NYHA functional class irrespective of their operative risk. No statistically significant difference among the subgroups in terms of functional class improvement was found (Figure 2).

### Survival

**Fig. 3:**
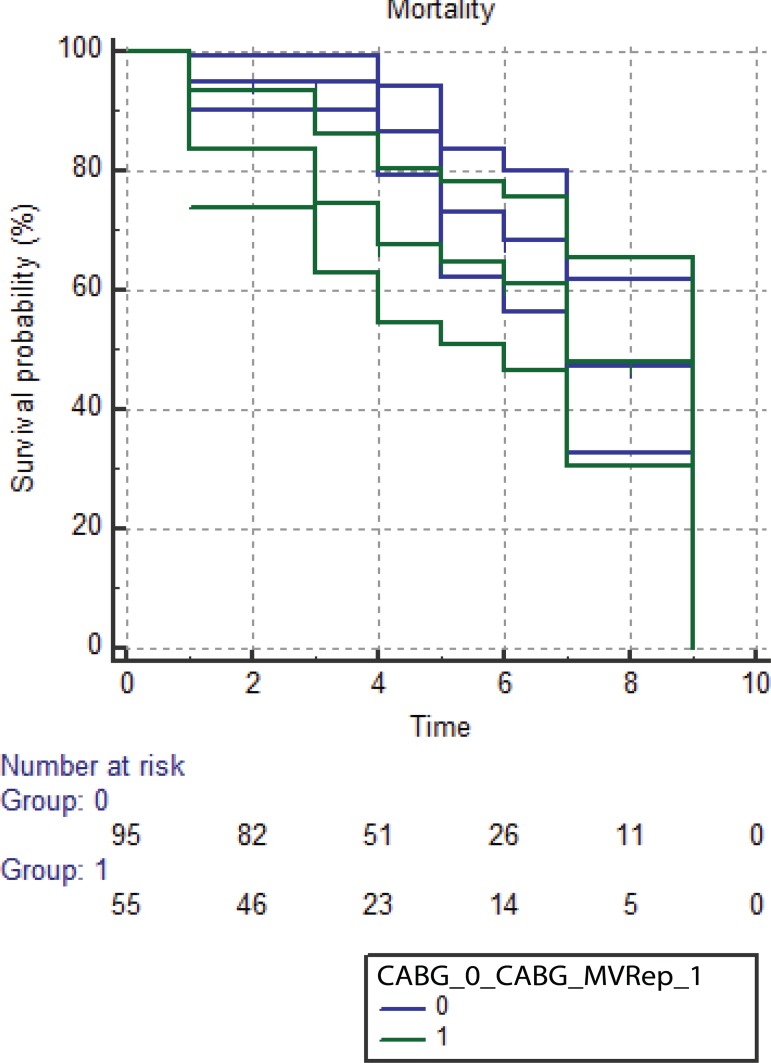
Kaplan-Meier survival curve between the off-pump coronary artery bypass grafting (OPCABG) and coronary artery bypass grafting (CABG)+ mitral valve repair (MVRep) groups.

In a mean follow-up period of five years, 35 patients of the entire cohort (group I - n=26, group II - n=9) died of non-cardiac cause and 12 (group I - n=7, group II - n=5) died of cardiac causes. The cumulative survival of OPCABG group was 63.2% and for the CABG + MVRep group was 54.5%. The 5-year survival was similar in both groups (Figure 3).

## DISCUSSION

This is probably the first study conducted in the elderly to compare the outcome of OPCABG *versus* CABG+MVRep for moderate CIMR. The routine use of antiplatelets, statins, beta blockers, and angiotensin converting enzyme inhibitors has greatly extended the age of CAD patients. It is now a common clinical scenario to see septuagenarians undergoing surgical myocardial revascularization. Even a healthy elderly is very different physiologically from a young as they have age-related thickening and stiffening of blood vessel walls leading to less elasticity, reduced diastolic function and poor effort tolerance compared to the young. They have reduced functional reserve of other organ systems compared to the young^[9]^. The incidence of cardiovascular disease increases as one's age advances. At 60-79 years, 70% will have cardiovascular disease of some form and 21% will have identifiable CAD^[10]^. This is reflected in our study as our cohort also has higher incidence of comorbidities.

The morbidity and mortality of surgical myocardial revascularization in the elderly have reduced significantly. Off-pump technique has added to the safety of surgical revascularization in the elderly. OPCABG has been shown to have reduced myocardial injury, reduced renal complications, reduced blood product usage and less neurological complications than on-pump CABG^[11]^. OPCABG can achieve complete revascularization comparable to on-pump CABG as shown in this study (mean number of grafts 3.14 vs. 3.21)^[12]^. In expert hands, OPCABG can also be safely accomplished in the presence of moderate CIMR.

CIMR involves incomplete closure of structurally normal mitral valve leaflet and is due to CAD that affects the LV geometry causing annular dilatation and tenting of the chordae tendineae^[13-15]^. Numerous studies have indicated association of CIMR with poor survival rates in CAD patients^[16,17]^. Surgical correction of moderate CIMR at the time of coronary revascularization is still an unresolved controversy^[18-21]^. Even in the recent ACC/AHA guidelines (2014), the level of evidence for surgical correction of moderate mitral regurgitation at the time of CABG is class IIB^[22]^. The addition of MVRep to CABG requires cardiopulmonary bypass with considerable length of aortic cross clamp time. Increased incidence of complications can be attributed to cardiopulmonary bypass in which, tissues and organs may suffer from regional malperfusion. The elderly with reduced functional reserve of many organ systems are more vulnerable to the complications of cardiopulmonary bypass. Extracorporeal circulation leads to various types of cellular injuries^[23]^. There was an increased incidence of supraventricular arrhythmias in the MVRep group, which may be due to the atrial incision for left atrial exposure. A similar trend of higher complications (24%) with CABG+MVRep group was observed by Smith et al.^[24]^. Randomized Ischemic Mitral Evaluation (RIME) trial^[25]^ also reported higher complication rates in MVRep group showing that adding MVRep should be weighed against the surgical morbidity and mortality risk the patient is exposed to. In our study, we have noted that CABG+MVRep group had high rate of early operative complications. We feel that the difference in our study is more pronounced as OPCABG group is completely excluded from the complications due to cardiopulmonary bypass.

We have documented in one of our earlier studies that mitral ring annuloplasty with CABG in a selected subgroup with severe CIMR provided mid-term improvement in mitral regurgitation grade and heart failure symptoms and it is a common practice now to repair severe CIMR at the time of revascularization^[26]^. There is no consensus about the benefits of repair in moderate CIMR. Chan et al.^[25]^ in RIME trial have reported improved functional capacity and increased LV reverse remodelling with MVRep and this finding is contradicted by the observations of Smith et al.^[24]^ who, in their trial had observed similar improvement in functional capacity and insignificant LV reverse remodelling and increased incidence of untoward events in the MVRep group. In our study, there was no significant difference in functional capacity and survival between the groups in a mean follow-up period of five years.

On subgroup analysis, we have found that patients with lower operative risk have better improvement in mitral regurgitation grade from CABG+MVRep as compared to OPCABG. We propose that they are probably the subgroup of elderly patients who can be considered for adding MVRep along with revascularization for moderate CIMR, but at the cost of a higher early postoperative complication rate.

Majority of the elderly patients have limited physical activity in their routine lifestyle and their life expectancy is limited. OPCABG can achieve complete revascularization, is less morbid, and provides comparable benefits in terms of functional class improvement and survival in the long term in comparison with CABG + MVRep for elderly patients with moderate CIMR.

## CONCLUSION

OPCABG is recommended for moderate CIMR in elderly patients with high operative risk. CABG+MVRep can be considered for moderate CIMR in elderly patients with low operative risk, but at the cost of higher early operative complications.

**Table:** 

Authors' roles & responsibilities	
AM	Substantial contributions to the conception or design of the work; or the acquisition; final approval of the version to be published
CA	Drafting the work or revising it critically for important intellectual content; final approval of the version to be published
VW	Drafting the work or revising it critically for important intellectual content; final approval of the version to be published
SS	Agreement to be accountable for all aspects of the work in ensuring that questions related to the accuracy or integrity of any part of the work are appropriately investigated and resolved; final approval of the version to be published
PS	Final approval of the version to be published
KP	Integrity of any part of the work are appropriately investigated and resolved; final approval of the version to be published
KS	Acquisition, analysis, or interpretation of data for the work; final approval of the version to be published
PS	Analysis, or interpretation of data for the work; final approval of the version to be published

## References

[1] Roques F, Nashef SA, Michel P, Gauducheau E, De Vincentiis C, Baudet E (1999). Risk factors and outcome in European cardiac surgery: analysis of
the EuroSCORE multinational database of 19030 patients. Eur J Cardiothorac Surg.

[2] Lamas GA, Mitchell GF, Flaker GC, Smith Jr SC, Gersh BJ, Basta L (1997). Clinical significance of mitral regurgitation after acute
myocardial infarction. Survival and Ventricular Enlargement
Investigators. Circulation.

[3] Grigioni F, Enriquez-Sarano M, Zehr KJ, Bailey KR, Tajik AJ (2001). Ischemic mitral regurgitation: long-term outcome and prognostic
implications with quantitative Doppler assessment. Circulation.

[4] Hickey MS, Smith LR, Muhlbaier LH, Harrell Jr FE, Reves JG, Hinohara T (1988). Current prognosis of ischemic mitral regurgitation. Implications
for future management. Circulation.

[5] Mack MJ, Pfister A, Bachand D, Emery R, Magee MJ, Connolly M (2004). Comparison of coronary bypass surgery with and without
cardiopulmonary bypass in patients with multivessel disease. J Thorac Cardiovasc Surg.

[6] Buffolo E, Lima RC, Salerno TA (2011). Myocardial revascularization without cardiopulmonary bypass:
historical background and thirty-year experience. Rev Bras Cir Cardiovasc.

[7] Gerola LR, Buffolo E, Jasbik W, Botelho B, Bosco J, Brasil LA (2004). Offpump versus on-pump myocardial revascularization in low-risk
patients with one or two vessel disease: perioperative results in a
multicenter randomized controlled trial. Ann Thorac Surg.

[8] Bonow RO, Carabello BA, Chatterjee K, Leon Jr AC, Faxon DP, Freed MD, American College of Cardiology, American Heart Association Task Force on Practice
Guidelines (2008). 2008 focused update incorporated into the ACC/AHA 2006 guidelines
for the management of patients with valvular heart disease: a report of the
American College of Cardiology/American Heart Association Task Force on
Practice Guidelines (Writing Committee to revise the 1998 guidelines for the
management of patients with valvular heart disease) Endorsed by the Society
of Cardiovascular Anesthesiologists, Society for Cardiovascular Angiography
and Interventions, and Society of Thoracic Surgeons. J Am Coll Cardiol.

[9] Karavidas A, Lazaros G, Tsiachris D, Pyrgakis V (2010). Aging and the cardiovascular system. Hellenic J Cardiol.

[10] Mozaffarian D, Benjamin EJ, Go AS, Arnett DK, Blaha MJ, Cushman M, American Heart Association Statistics Committee and Stroke
Statistics Subcommittee (2015). Heart disease and stroke statistics-2015 update: a report from
the American Heart Association. Circulation.

[11] Al-Ruzzeh S, George S, Yacoub M, Amrani M (2001). The clinical outcome of off-pump coronary artery bypass surgery
in the elderly patients. Eur J Cardiothorac Surg.

[12] Al-Ruzzeh S, Nakamura K, Athanasiou T, Modine T, George S, Yacoub M (2003). Does off-pump coronary artery bypass (OPCAB) surgery improve the
outcome in high-risk patients?: a comparative study of 1398 highrisk
patients. Eur J Cardiothorac Surg.

[13] Komeda M, Glasson JR, Bolger AF, GT 2nd Daughters, MacIsaac A, Oesterle SN (1997). Geometric determinants of ischemic mitral
regurgitation. Circulation.

[14] Watanabe N, Ogasawara Y, Yamaura Y, Wada N, Kawamoto T, Toyota E (2005). Mitral annulus flattens in ischemic mitral regurgitation:
geometric differences between inferior and anterior myocardial infarction: a
real-time 3-dimensional echocardiographic study. Circulation.

[15] Agricola E, Oppizzi M, Maisano F, De Bonis M, Schinkel AF, Torracca L (2004). Echocardiographic classification of chronic ischemic mitral
regurgitation caused by restricted motion according to tethering
pattern. Eur J Echocardiogr.

[16] Castleberry AW, Williams JB, Daneshmand MA, Honeycutt E, Shaw LK, Samad Z (2014). Surgical revascularization is associated with maximal survival in
patients with ischemic mitral regurgitation: a 20-year
experience. Circulation.

[17] Grigioni F, Detaint D, Avierinos JF, Scott C, Tajik J, Enriquez-Sarano M (2005). Contribution of ischemic mitral regurgitation to congestive heart
failure after myocardial infarction. J Am Coll Cardiol.

[18] Milano CA, Daneshmand MA, Rankin JS, Honeycutt E, Williams ML, Swaminathan M (2008). Survival prognosis and surgical management of ischemic mitral
regurgitation. Ann Thorac Surg.

[19] Kang DH, Kim MJ, Kang SJ, Song JM, Song H, Hong MK (2006). Mitral valve repair versus revascularization alone in the
treatment of ischemic mitral regurgitation. Circulation.

[20] Wong DR, Agnihotri AK, Hung JW, Vlahakes GJ, Akins CW, Hilgenberg AD (2005). Long-term survival after surgical revascularization for moderate
ischemic mitral regurgitation. Ann Thorac Surg.

[21] Diodato MD, Moon MR, Pasque MK, Barner HB, Moazami N, Lawton JS (2004). Repair of ischemic mitral regurgitation does not increase
mortality or improve long-term survival in patients undergoing coronary
artery revascularization: a propensity analysis. Ann Thorac Surg.

[22] Nishimura RA, Otto CM, Bonow RO, Carabello BA, JP 3rd Erwin, Guyton RA, American College of Cardiology/American Heart Association Task Force
on Practice Guidelines (2014). 2014 AHA/ACC guideline for the management of patients with
valvular heart disease: a report of the American College of
Cardiology/American Heart Association Task Force on Practice
Guidelines. J Am Coll Cardiol.

[23] Wan S, LeClerc JL, Vincent JL (1997). Inflammatory response to cardiopulmonary bypass: mechanisms
involved and possible therapeutic strategies. Chest.

[24] Smith PK, Puskas JD, Ascheim DD, Voisine P, Gelijns AC, Moskowitz AJ (2014). Surgical treatment of moderate ischemic mitral
regurgitation. N Engl J Med.

[25] Chan KM, Punjabi PP, Flather M, Wage R, Symmonds K, Roussin I, RIME Investigators (2012). Coronary artery bypass surgery with or without mitral valve
annuloplasty in moderate functional ischemic mitral regurgitation: final
results of the Randomized Ischemic Mitral Evaluation (RIME)
trial. Circulation.

[26] Malhotra A, Sharma P, Garg P, Bishnoi A, Kothari J, Pujara J (2014). Ring annuloplasty for ischemic mitral regurgitation: a single
center experience. Asian Cardiovasc Thorac Ann.

